# Preference of Virtual Reality Games in Psychological Pressure and Depression Treatment: Discrete Choice Experiment

**DOI:** 10.2196/34586

**Published:** 2023-01-16

**Authors:** Shan Jin, Zijian Tan, Taoran Liu, Sze Ngai Chan, Jie Sheng, Tak-hap Wong, Jian Huang, Casper J P Zhang, Wai-Kit Ming

**Affiliations:** 1 The Thrust of Computer Media and Art The Hong Kong University of Science and Technology (Guangzhou) Guangzhou China; 2 Department of Public Health and Preventive Medicine, School of Medicine Jinan University Guangzhou China; 3 Department of Infectious Diseases and Public Health Jockey Club College of Veterinary Medicine and Life Sciences City University of Hong Kong Hong Kong China (Hong Kong); 4 Department of Obstetrics and Gynaecology, First Affiliated Hospital of Jinan University Guangzhou China; 5 Department of Epidemiology and Biostatistics, School of Public Health Imperial College London London United Kingdom; 6 School of Public Health The University of Hong Kong Hong Kong China (Hong Kong)

**Keywords:** virtual reality, discrete choice experiment, college student, depression therapy

## Abstract

**Background:**

Virtual reality (VR) can be used to build many different scenes aimed at reducing study-related stress. However, only few academic experiments on university students for preference testing have been performed.

**Objective:**

This study aims to assess the preference of VR games for stress and depression treatment using a discrete choice experiment (DCE).

**Methods:**

A total of 5 different attributes were selected based on the depression therapy parameters and attributes related to VR: (1) treatment modality; (2) therapy duration; (3) perceived remission rate; (4) probability of adverse events; and the (5) monthly cost of adding treatment to a discrete choice experiment. By comparing different attributes and levels, we could draw some conclusions about the depression therapy testing preference for university students; 1 university student was responsible for VR scene development and 1 for participant recruitment.

**Results:**

The utility value of different attributes for “0% Probability of adverse events” was higher than others (99.22), and the utility value of VR treatment as the most popular treatment method compared with counseling and medicine treatment was 80.95. Three parameter aspects (different treatments for depression) were statistically significant (P<.001), including “0%” and “50%” of “Probability of adverse events” and “¥500” (a currency exchange rate of ¥1 [Chinese yuan]=US $0.15 is applicable) of “The monthly cost of treatment.” Most individuals preferred 12 months as the therapy duration, and the odds ratio of “12 months” was 1.095 (95% CI 0.945-1.270) when compared with the reference level (6 months). Meanwhile, the cheapest price (¥500) of depression therapy was the optimum choice for most students.

**Conclusions:**

People placed great preference on VR technology psychological intervention methods, which indicates that VR may have a potential market in the treatment of psychological problems. However, adverse events and treatment costs need to be considered. This study can be used to guide policies that are relevant to the development of the application of VR technology in the field of psychological pressure and depression treatment.

## Introduction

According to a report provided by World Health Organization [1], the incidence of depression accounts for about 4.4% of the total world population [2,3]. A detailed report [4] specifically about the prevalence of depression among Chinese individuals was recently published [5]. The China Mental Health Survey estimated that more than 95 million patients have depression in China: 83% of the patients with depression were reported to be older than 35 years old, and 65% of the patients were female [6]. Among Chinese university students, the overall prevalence of depression was 28.4% (n=185,787), and the percentage of depressed individuals was reported to be high [7].

Depression is the emotional expression of a state of ego-helplessness and ego-powerlessness to live up to certain strongly maintained narcissistic aspirations [8]. Individuals with depressive disorders usually feel sad, lonely, or irritable, and in most cases, they are in a bad mood [9]. People with depression usually experience at least five of the following 9 characteristics: (1) people feel sad and unhappy; (2) people have less interest in doing things; (3) individuals do not follow a diet, but their weight losses are obvious; (4) their weights increase, or their appetites change; (5) they suffer insomnia or drowsiness; (6) some people have mental agitation; (7) they feel that life is meaningless, or they feel guilty; (8) they have difficulty concentrating; or (9) they have suicidal thoughts [10,11].

Three categories offer reasons that individuals have this illness: (1) the influence of environmental factors (eg, abuse, criticism, or neglect in childhood); (2) the genetic factors (individuals whose close relatives have been diagnosed with depression are at an increased risk); or (3) the effects of hormones, such as serotonin and norepinephrine [12-14].

At present, the 3 main therapy methods to tackle this issue are drug treatment (antidepressants, antipsychotics), psychotherapy (psychotherapy consultation), and phototherapy (usually suitable for dealing with seasonal depression). Other alternative therapy methods are Chinese herbal medicine, exercise, meditation, self-relaxation, and others. Some antidepressants have side effects, and the cost of psychotherapy is high [15-17].

Virtual reality (VR) technology has *3I* characteristics, namely, immersion, interaction, and imagination [18,19]. In the previous research on VR, as it relates to therapy for depression, a new concept of VR exposure therapy had been used in the treatment of anxiety and depression. The growing body of literature suggests that VR exposure therapy is a successful tool for relieving stress caused by psychological problems [20-22]. For example, the VR gaming experience could be used in conjunction with exercise, using games such as VirZoom, in which a VR exercise bike is compatible with most VR headsets [23,24]. The participant could wear the headset and alleviate stress and depression using the game [25].

According to the literature review, DCE is not widely applied in the field of VR technology. Research on the uses of VR technology has only covered a few areas, such as fire emergency exit testing [26], willingness-to-pay services research [27], and the testing of pedestrian behavior models [28]. By contrast, there are studies on depression and DCE. Lokkerbol et al [29] demonstrated how different components of depression treatment could impact patients’ preferences. The study conducted by van Loenen et al [30] showed that DCE could support the preference of genetic testing for the user’s willingness-to-pay and help improve depression therapy. Actually, some research suggests that the VR technology is useful in treating depression [22-25]. For example, the result from Schleider et al [31] revealed that using immersive technology, such as a VR headset, may be an efficient strategy for reducing adolescent depressive symptoms. However, to our knowledge, there is no research that investigates the participants’ preference regarding using VR technology in treating depression. There is still a big gap in the application of VR technology in DCE, and our experiments could help in this field. The purpose of this experiment is to investigate the preference for VR technology in psychological pressure relief and depression treatment for college students.

## Methods

### Study Sample

The study was conducted at 2 comprehensive universities in China, Jinan University in Guangzhou and the University of Electronic Science and Technology of China (UESTC) in Chengdu. We recruited our study participants from different majors at the 2 universities. Inclusion criteria included 3 parameters: (1) at least 18 years old; (2) interested in their mental state or with depressive disorders/episodes; and (3) interested in using VR technology to improve mental health problems. Those who did not agree to participate in the study and were suffering from major diseases other than depression and other mental illnesses were excluded.

### Process

**Figure 1:**
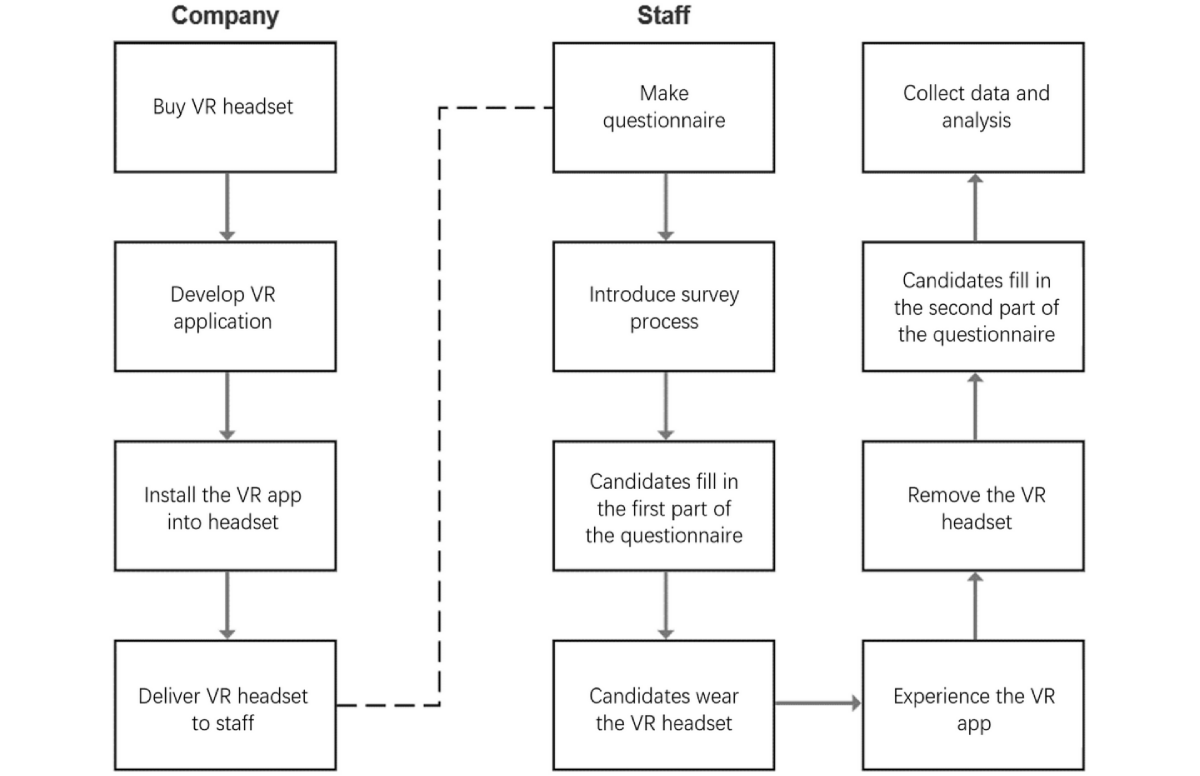
The process of conducting the experiment. VR: virtual reality.

Figure 1 illustrates our research process. Two groups of students from different areas were enrolled in this study. Students in group 1 came from the UESTC in Chengdu, and students in group 2 came from Jinan University in Guangzhou. The students from the UESTC needed to borrow a VR device as they did not have a VR headset to support app development.

### Discrete Choice Experiment

Discrete choice experiment (DCE) is a utility method in the field of economics [32]. The theory is not only applicable to economics but is also widely used in other disciplines, such as in industries, education, software, and medical care [33,34]. Medical applications include prenatal diagnostic testing and myocardial infarction diagnostic testing [35]. We conducted the questionnaire survey and VR game experience and then collected the corresponding statistical data based on the results that were collected [36,37].

### VR Platform

Our devices were a Pico Neo 2 (Sichuan Shuyun Technology Co., Ltd) with a head display, earphones, handle, connecting cable, and other accessories. The VR headset also has some disadvantages: when people wear this device for a long time to play games, it may cause dizziness, nausea, eye fatigue, blurred vision, and other problems [38-40]. However, those negative influences will decrease if users just wear the headset for several minutes.

### Virtual Reality Applications

**Figure 2:**
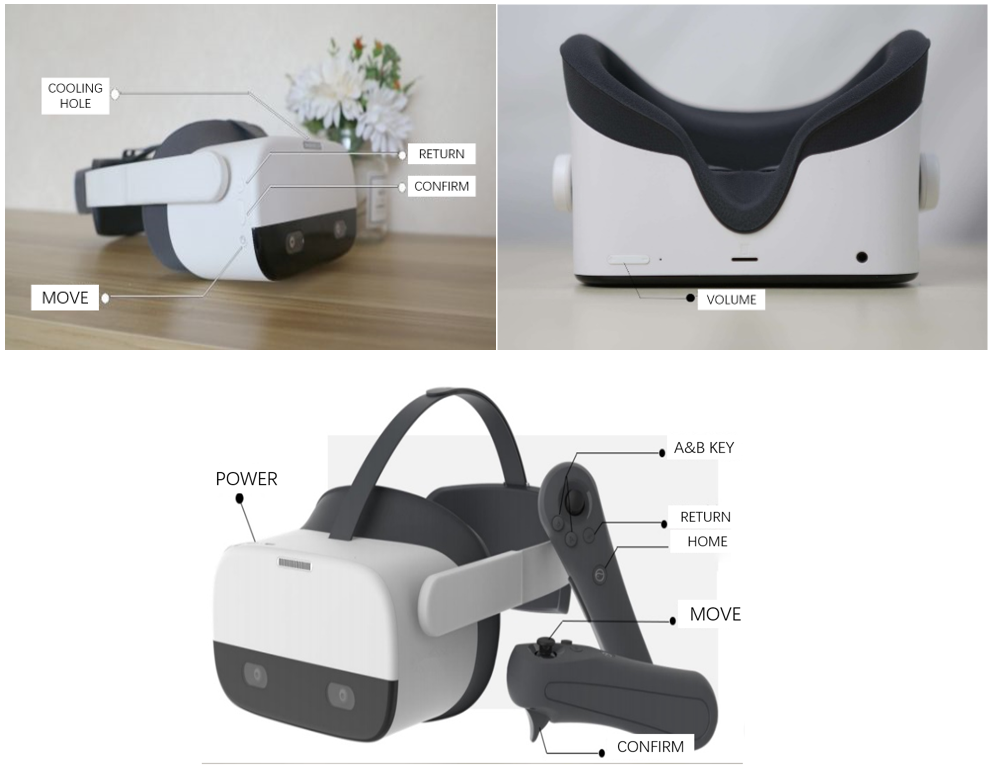
Display of handle, headset, and function key in the device.

**Figure 3:**
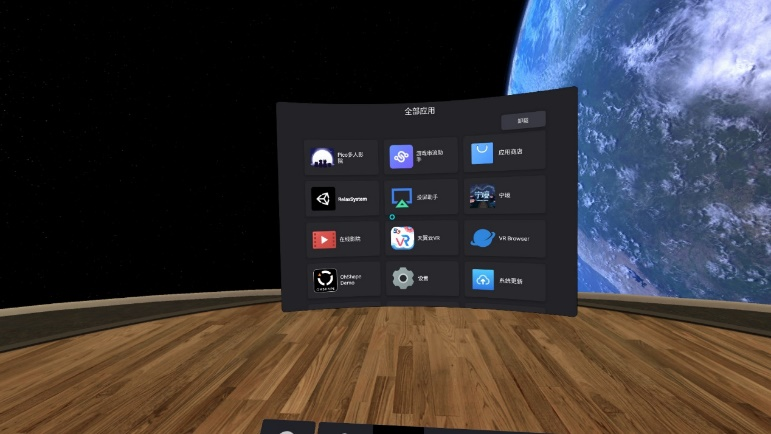
Application Panel for Pico Neo 2 (Sichuan Shuyun Technology Co., Ltd).

Figures 2 and 3 demonstrate the VR device and application panel, respectively. It is obvious that scholars have previously performed some research experiments on static scene VR applications; thus, we selected 2 dynamic and interactive applications in this experiment: (1) VR physical and mental relaxation system and (2) VR music comfort system.

**Figure 4:**
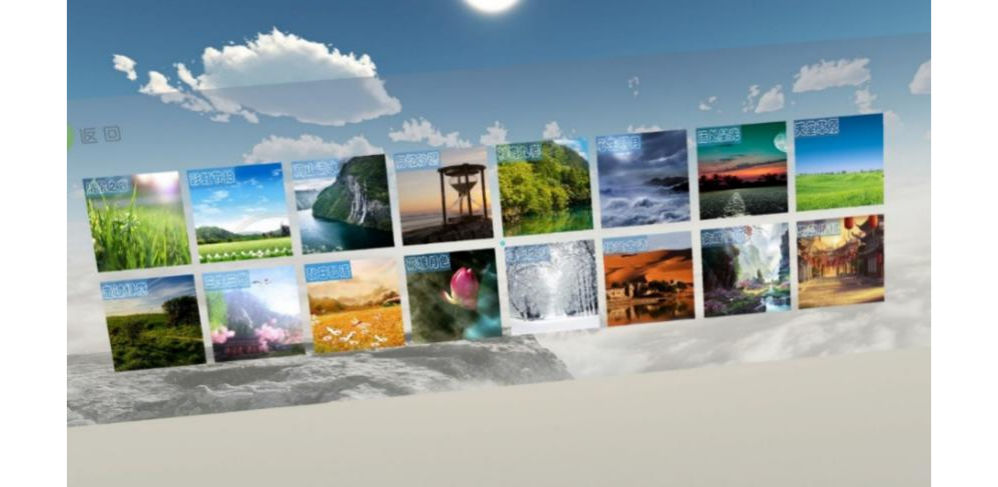
Scene selection interface (includes 16 various scenes, such as historical city, beautiful mountain, waterfall, and peaceful lake).

**Figure 5:**
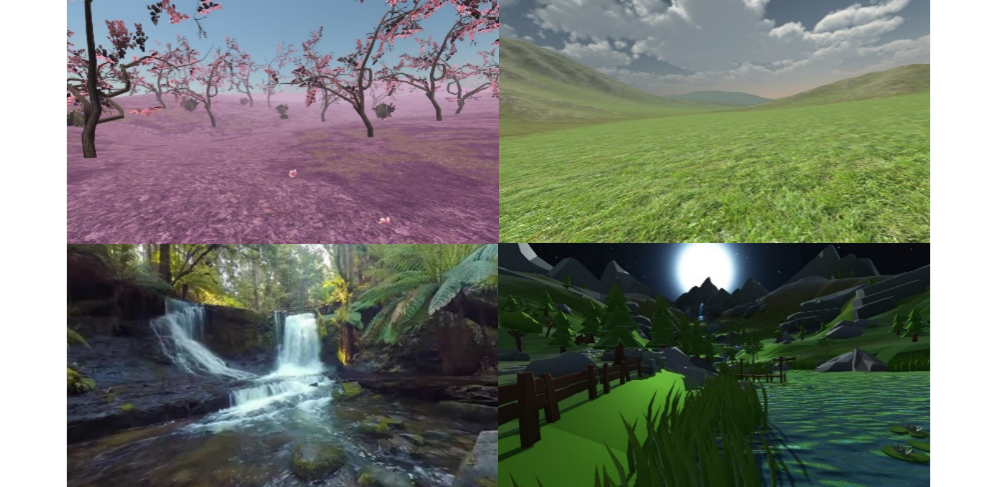
Scene examples (the first picture illustrates peach blossom, the second scene is a lawn on top of a mountain, the third is a 360° video that we could watch at different angles in the virtual world, and the fourth is a peaceful scene under the moon at night).

The VR physical and mental relaxation system needs participants to cooperate with the virtual professional psychologist’s guidance into scenes to help them correctly and quickly adjust to negative emotions, such as psychological anxiety and tension. First, this system could provide 16 3D realistic natural sceneries (Figures 4 and 5) so that patients can enjoy the beauty of nature without leaving home with the aim of relieving anxiety and maintaining physical and mental health for themselves. Second, based on the logic of relaxation therapy, it can provide a psychotherapist’s voice as a relaxation guide to help patients correctly relax their body and mind, and it allows them to become immersed in the atmosphere of nature to quickly eliminate negative emotions and adjust their mentality.

We designed a “VR music comfort system,” which included dozens of psychological medical music approaches. Through comfortable music activities, this system could trigger positive experiences in physiology to promote perception, memory, and arousal. This application’s key features are as follows: users can choose different scenes according to their preferences and can listen to music to relax. At the same time, users can also turn on the voice guidance function to receive guidance regarding depression and relaxation. The key step here is the user interface design because we must make sure participants could listen to the music from different VR buttons and experience the application without failure. Unity 3D and Visual Studio were used to create the user interface design, and we must make sure participants can wear the headset and click different scenes from the VR buttons correctly. Then, they can listen to music and travel to different environments in VR. At the same time, this system also integrates a positive music interaction system with the aim of promoting people’s physical and mental health; thus, music interactive scene training is carried out in the virtual world to relieve emotions [41,42].

### Attributes and Levels

In this paper, for the DCE attribute and the level design, we conducted a focus group with the professors in medicine at Jinan University. We came up with 5 attributes and levels. In addition, for details on the setting of the levels, we performed a literature search. There are some studies on the perceived remission rate and the incidence of adverse reactions regarding the selection of depression indicators. Different countries and experts have their own indicator considerations. Possible attributes were identified from a panel of experienced chief physicians from Jinan University. Finally, 5 different attributes were selected: (1) treatment modality, (2) therapy duration, (3) perceived remission rate, (4) probability of adverse events, and (5) the monthly cost of treatment. Based on the academic research experiments for depression therapy, we divided the attributes into several independent parameters concerning therapy duration [43,44], cost [45-48], perceived remission rate [49-55], and probability of adverse events [56-60] with the aim of building different intervals for real life. To eliminate the influence of dimensions between different attributes, the average utility values of all attribute levels were measured using the utility scaling method with zero-centered differences. Attributes and levels are shown in Table 1.

**Table 1:** Attributes and levels.

Attributes	Levels of attributes
Treatment modality	kL1 Medication only
	L2 Counseling only
	L3 VRa only
Therapy duration	L1 6 months
	L2 12 months
	L3 18 months
	L4 Lifetime
Perceived remission rate	L1 35%
	L2 40%
	L3 45%
	L4 50%
Probability of adverse events	L1 0%
	L2 30%
	L3 50%
The monthly cost of treatment	L1 ¥500^b^
	L2 ¥1000
	L3 ¥1500
	L4 ¥2000
	L5 ¥2500

^a^VR: virtual reality.

^b^A currency exchange rate of ¥1 (Chinese yuan)=US $0.15 is applicable.

### Questionnaire

This questionnaire is collected from the online questionnaire platform. It includes 4 parts: (1) basic information (5 questions: gender, education level, the impact of depression on health, level of understanding of depression, and level of understanding of VR technology); (2) the first DCE experiment; (3) VR experience evaluation feedback (5 questions: effective treatments for depression, the function of this VR app in therapy, defects of VR in psychotherapy, the best time for VR for psychotherapy, and VR scenes preferred to experience); and (4) the second DCE experiment. In the beginning, the users should complete the basic information collection step; then, they will fill in the first part of the DCE questionnaire. After finishing these sections, they will be asked to wear the VR headset and experience the apps. They will then need to finish the VR experience evaluation feedback part and the second DCE questionnaire sequence. Brief introductions for participants to acquire related knowledge at the beginning of each part, such as VR’s introduction, DCE procedure, and the meaning of the probability of adverse events, will be included. The duration of the VR applications will be around 5 minutes. Based on completing the questionnaire prior to VR application and pretesting the VR, participants were allowed 20 minutes to complete the research. An interactive design was devised; when the cursor or fingers touch the nouns (perceived remission rate, probability of adverse events), volunteers will see the introduction around the concepts and gain some useful information about them [35,36].

The first part of the DCE was conducted before the participants viewed the VR scenes, and the second part of the DCE was conducted after the participants had viewed the VR scenes. This research compared the 2 different DCE parts to understand whether VR could produce different effects on different participants. Each DCE contained 6 multiple-choice questions with 3 different options: (1) intervention mode 1, (2) intervention mode 2, and (3) other ways. The example questions of DCE are shown in Table 2. For the DCE part, the same DCE design (ie, a random draw of the set of questionnaires from the pool with the same attributes and levels) was used for the “before-and-after” comparison. In this study, we compared the preference “before-and-after” of the participants. Our discussion about the 2 DCE parts has different questions in the “before-and-after” comparison because we subtracted various parameters from the 5 attributes in every DCE’s part to discover the user’s preference for depression therapy before and after experiencing the VR applications. Some parts of the DCE design are referred to in Ryan and Gerard’s [33] experiment, which provides better guidance on disease prevention and treatment using DCE.

**Table 2:** An example of discrete choice experiment question.

Attributes	Treatment A	Treatment B	Neither
Treatment modality	VR^a^ only	Medication only	
Therapy duration	12 months	18 months	Neither
Perceived remission rate, %	35	40	
Probability of adverse events, %	0	50	
The monthly cost of treatment (¥)^b^	500	2000	

^a^VR: virtual reality.

^b^A currency exchange rate of ¥1 (Chinese yuan)=US $0.15 is applicable.

### Statistical Analysis

#### Latent Class Analysis Model

Latent class analysis was used to interpret the DCE data. The conditional logit regression model was used to quantify the correlation between the participant’s choice and the attribute levels of various test profiles for which the choices were used as the dependent variable and the attribute levels of tests as covariates. The levels for the 5 attributes were coded based on effects. The conditional logic model makes statistical inferences based on the weights of the respondents’ preferences for various attributes and levels in the questionnaire. The resulting positive and negative coefficients after the regression analysis indicated the direction of the respondent’s preference for a given attribute level. Based on the importance and magnitude of the coefficients, the marginal rate of substitution between each attribute and the relative importance of the attributes was calculated, which could measure the willingness to accept a trade-off among different options of respondents. These marginal rate of substitution values allowed for the comparison of different attributes using a common scale [61], and the odds ratios (ORs) of respondents’ preferences for different attribute levels were also reported. Sawtooth (Sawtooth Software, Inc.) was used to run the coefficients of all attributes and SEs, and *t* ratios to calculate the P values. For the P value of all levels of attributes, we assumed that if the P value of a level was <.05, this level was then statistically significant; when the P value of a level was <.001, this level was considered highly statistically significant.

#### Latent Class Model

A latent class analysis was conducted to identify correlations among explicit variables, create the fewest number of classes, and achieve local independence. According to Greene and Hensher [62], this method uses a semiparametric approach to model the correlation structure of the data and identify classes that are more homogeneous in terms of variance structure. This method of analysis can be used to sort individuals into a set of classes with a certain segmented size and scale, and different effects of each class were estimated for different attributes. In addition, this method will help us to measure the differences and similarities of preference across classes of respondents. Akaike information criterion 3 and Bayesian information criteria (AIC3 and BIC, respectively) were used as the main criteria, and after the latent class model was created, the resulting data were classified into the appropriate latent classes.

#### Software

All statistical analyses were performed using Sawtooth Lighthouse Studio (SSI Web version 9.11.0; Sawtooth Software, Inc.).

### Ethics Approval

This study was approved by the Medical Ethics Committee of Jinan University (JNUKY-2021-004).

## Results

### Survey Participants

A total of 154 people were surveyed, among which 114 completed all the questionnaire contents (response rate 74%). The summary of demographic information and the suggestions and preferences of participants after using VR are shown in Table 3.

**Table 3:** Summary of demographic information, suggestions, and preferences of participants.

Items			Total (N=114), n (%)		Medical students (n=71), n (%)		Nonmedical students (n=43), n (%)		P value
**Gender**									.84
	Male	65 (57.0)		43 (60.6)		22 (51.2)			
	Female	49 (42.9)		28 (39.4)		21 (48.8)			
**Education level**									.04^a^
	College degree	13 (11.4)		6 (8.5)		7 (16.3)			
	Bachelor	92 (80.7)		57 (80.3)		35 (81.4)			
	Master	8 (7.0)		7 (9.9)		1 (2.3)			
	Doctoral	1 (0.9)		1 (1.4)		0 (0.0)			
**The impact of depression on health**									.62
	No effect	6 (5.3)		5 (7.0)		1 (2.3)			
	Affects only emotions	22 (19.3)		14 (19.7)		8 (18.6)			
	Has a certain impact	29 (25.4)		16 (22.5)		13 (30.2)			
	Has a serious impact	57 (50.0)		36 (50.7)		21 (48.8)			
**Level of understanding of depression**									.18
	Not clear	14 (12.3)		8 (11.3)		6 (14.0)			
	Slightly heard	56 (49.1)		35 (49.3)		21 (48.8)			
	General understanding	38 (33.3)		24 (33.8)		3 (7.0)			
	Understand completely	6 (5.3)		4 (5.6)		4 (9.3)			
**Level of understanding of** **virtual reality**									.20
	Not clear	17 (14.9)		8 (11.3)		9 (20.9)			
	Slightly heard	64 (56.1)		43 (60.6)		21 (48.8)			
	General understanding	23 (20.2)		12 (16.9)		11 (25.6)			
	Understand completely	10 (8.8)		8 (11.3)		2 (4.7)			
**Effective treatments for depression**									.54
	Psychological consultation	74 (64.9)		46 (64.8)		28 (65.1)			
	Medicine treatment	83 (72.8)		49 (69.0)		34 (79.1)			
	Movement regulation	57 (50.0)		36 (50.7)		21 (48.8)			
	Self-regulation	67 (58.8)		38 (53.5)		29 (67.4)			
	Virtual reality treatment	44 (38.6)		21 (29.6)		23 (53.5)			
**Function of this virtual reality app in therapy**									.20
	Relieve stress	65 (57.0)		36 (50.7)		29 (67.4)			
	Divert attention	71 (62.3)		45 (63.4)		26 (60.5)			
	Satisfy vanity	20 (17.5)		12 (16.9)		8 (18.6)			
	No function or have other function	14 (12.3)		12 (16.9)		2 (4.7)			
**Defects of virtual reality in psychotherapy**									.86
	Uncomfortable wearing	20 (17.5)		12 (16.9)		8 (18.6)			
	Technology is immature	35 (30.7)		22 (31.0)		13 (30.2)			
	Expensive	29 (25.4)		17 (23.9)		12 (27.9)			
	Not easy to operate	16 (14.0)		8 (11.3)		8 (18.6)			
**The best time for virtual reality for psychotherapy**									.47
	Immediately aware of stress	15 (13.2)		10 (14.1)		5 (11.6)			
	When depression is diagnosed	27 (23.7)		14 (19.7)		13 (30.2)			
	During psychotherapy	32 (28.1)		17 (23.9)		15 (34.9)			
	Experience at any time in daily life	26 (22.8)		18 (25.4)		8 (18.6)			
**Virtual reality scenes preferred to experience**									.006^a^
	Quiet real virtual environment	64 (56.1)		37 (52.1)		27 (62.8)			
	Strange real virtual environment	43 (37.7)		23 (32.4)		20 (46.5)			
	Help yourself recall the past	52 (45.6)		26 (36.6)		26 (60.5)			
	Experience the good things of the past	53 (46.5)		28 (39.4)		20 (46.5)			
	Watching movies and television shows	34 (29.8)		14 (19.7)		16 (37.2)			
	Play simple mini-games	31 (27.2)		15 (21.1)		12 (27.9)			
	The experience that dreams come true	25 (21.9)		13 (18.3)		29 (67.4)			
	Play exciting and fun big games	25 (21.9)		16 (22.5)		12 (27.9)			
	Other	14 (12.3)		12 (16.9)		9 (20.9)			

^a^P<.05 indicates statistical significance.

For the VR experience, evaluation feedback results showed that most individuals preferred the choice of VR helmet weight, comfort, and application contents. Students also thought that VR relieves stress and diverts attention. Furthermore, students would choose the immersive scenes, memorable places from their childhood, and beautiful fantasies of the virtual world for the VR depression therapy after the VR experience.

### Attributes’ Levels and Utility Report

In the study, the utility scaling method with zero-centered differences was used to measure the average utility values of all the attribute levels and the utility gap between different treatment methods; additionally, the probability of adverse events was overt. For example, the utility of VR treatment alone was 80.95, whereas it was −42.66 and –38.30 for the attributes “Medication only” and “Psychological counseling,” respectively; for 30% probability of adverse events, the utility value was –13.81%, whereas for 50% adverse reactions, the utility value was –85.41%.

To eliminate the influence of dimensions between different attributes, the average utility values of all attribute levels were measured using the User-based Security Model with zero-centered differences. The highest utility levels of the 5 hypothesized attributes were “VR only” (of “Treatment modality”), “12 months” (of “Therapy duration”), “40%” (of “Perceived remission rate”), “0%” (of “Probability of adverse events”), and “¥500” (of “The monthly cost of treatment”; a currency exchange rate of ¥1 [Chinese yuan]=US $0.15 is applicable). The most important attribute was “Probability of adverse events,” indicating that most respondents ranked that attribute the highest (Table 4).

**Table 4:** The utility report of different attributes.

Attributes and levels			Utility
**Treatment modality**			
	VR^a^ only	80.95	
	Medication only	–42.66	
	Counseling only	–38.30	
**Therapy duration**			
	6 months	6.62	
	12 months	23.24	
	18 months	5.72	
	Lifetime	–35.57	
**Perceived remission rate**			
	35%	0.22	
	40%	10.81	
	45%	–5.35	
	50%	0.45	
	55%	–6.14	
**Probability of adverse events**			
	0%	99.22	
	30%	–13.81	
	50%	–85.41	
**The monthly cost of treatment^b^**			
	¥500	69.44	
	¥1000	16.18	
	¥1500	3.64	
	¥2000	–42.71	
	¥2500	–46.55	
**None**			
	N/A^c^	–336.97	

^a^VR: virtual reality.

^b^A currency exchange rate of ¥1 (Chinese yuan)=US $0.15 is applicable.

^c^N/A: not applicable.

### Logit Result of DCE and Attributes’ Percentage Importance

**Figure 6:**
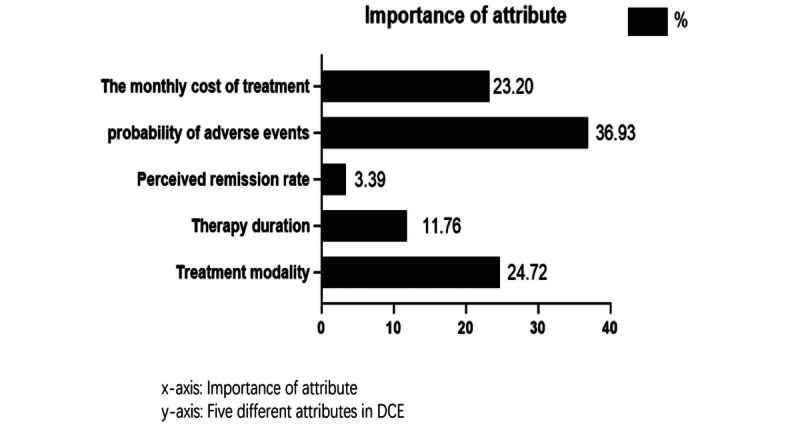
Importance of attributes. DCE: discrete choice experiment.

As is presented in Figure 6, the percentage importance of the attribute “Probability of adverse events” was the highest, which shows that people reported being most concerned about the probability of adverse events. The attributes “Treatment modality” and “The monthly cost of treatment” ranked second and third, respectively.

The results of the logit analysis of all attribute levels are presented in Table 5. For treatment modality, the coefficient of the level “VR only” was positive, which indicated that the level “VR only” was positively correlated with people’s preferences and use. In addition, the coefficients of the levels “6 months,” “12 months,” and “18 months” of “Therapy duration” were positive, and “lifetime” was negative. It is clear that people showed a preference for shorter periods of therapy, but not necessarily for the shortest one. For the attribute “Therapy duration,” people were more inclined to choose the level of “12 months.” For the levels of other attributes, people preferred the lower probability of adverse events and lower treatment costs. However, for the attribute “Perceived remission rate,” people unexpectedly preferred a lower perceived remission rate rather than the “55%” option. A reasonable explanation for this phenomenon is that individuals with various degrees of histories, genders, and other backgrounds would have a different understanding of this concept (perceived remission rate). In addition, some of the participants neglected the tips around this concept in our questionnaire. We found that “VR only,” “Medication only,” and “Counseling only” for “Diagnosis methods” were highly statistically significant (P<.001 for all), “0%” and “50%” of “Probability of adverse events” were extremely statistically significant (P<.001), and “¥500” of “The monthly cost of treatment” was also statistically significant (P<.001).

Taking the level “VR only” of the “Treatment modality” attribute as the reference, the ORs of “Medication only” and “Counseling only” were 0.509 (95% CI 0.452-0.573) and 0.521 (95% CI 0.462-0.588), respectively. The OR of “12 months” of “Therapy duration” was 1.095 (95% CI 0.945-1.270) with the level “6 months” as the reference. ORs for “30%” and “50%” of the “Probability of adverse events” attribute were 0.539 and 0.364, respectively, with the level “0%” as the reference (95% CI 0.480-0.605 and 0.323-0.412, respectively). All attribute levels for “The monthly cost of treatment” were <1, which indicated that the preference weights declined with increasing medical fees.

**Table 5:** The result of logit analysis of preference in general (N=114).

Attribute and levels			Coefficient (SE)		P value		Odds ratio (95% CI)
**Treatment modality**							
	VR^a^ only	0.44257 (0.06067)		<.001^b^		Reference	
	Medication only	–0.23321 (0.06124)		<.001^b^		0.509 (0.452-0.573)	
	Counseling only	–0.20936 (0.06126)		<.001^b^		0.521 (0.462-0.588)	
	6 months	0.03617 (0.07616)		.64		Reference	
	12 months	0.12704 (0.07548)		.09		1.095 (0.945-1.270)	
	18 months	0.03126 (0.07691)		.68		0.995 (0.856-1.157)	
	Lifetime	–0.19447 (0.07715)		<.05		0.794 (0.683-0.924)	
**Perceived remission rate**							
	35%	0.00123 (0.0894)		.99		Reference	
	40%	0.05912 (0.08968)		.51		1.060 (0.889-1.263)	
	45%	–0.02924 (0.08919)		.74		0.970 (0.814-1.155)	
	50%	0.00248 (0.08993)		.98		1.001 (0.839-1.194)	
	55%	–0.03359 (0.09085)		.71		0.966 (0.808-1.154)	
**Probability of adverse events**							
	0%	0.54244 (0.06096)		<.001^b^		Reference	
	30%	–0.07549 (0.05907)		.20		0.539 (0.480-0.605)	
	50%	–0.46695 (0.06215)		<.001^b^		0.364 (0.323-0.412)	
**The monthly cost of treatment**							
	¥500^c^	0.37962 (0.08936)		<.001^b^		Reference	
	¥1000	0.08844 (0.08881)		.32		0.747 (0.628-0.889)	
	¥1500	0.01993 (0.09042)		.83		0.698 (0.585-0.833)	
	¥2000	–0.2335 (0.09043)		.01^d^		0.542 (0.454-0.647)	
	¥2500	–0.25448 (0.08962)		.005^d^		0.530 (0.445-0.632)	
**None**							
	N/A^e^						

^a^VR: virtual reality.

^b^P<.001 indicates highly statistical significance.

^c^A currency exchange rate of ¥1 (Chinese yuan)=US $0.15 is applicable.

^d^P<.05 indicates statistical significance.

^e^N/A: not applicable.

### Latent Class Analysis Result

We compared these potential models, selected the model that maximized the area under the receiver–operating characteristic curve, and minimized the AIC or BIC to compensate for model complexity [63]. According to AIC, 5 classes should have been the best choice for our model. By contrast, BIC favored the 2-class option with the lowest BIC. Under such circumstances, we compared A BIC, that is, sample size–adjusted BIC, which involved sample size values. After comparison, the 2-class option had the lowest value of A BIC. Therefore, the most suitable number of latent classes in our model was 2 (Tables 6 and 7). First, all 114 respondents were divided into 2 classes with segment sizes of 77 (67.5%) and 37 (32.5%), respectively. The average maximum membership probability was around 0.87, and the percent certainty was 26.17, which is relatively low, indicating that a low level of uncertainty according to the respondents is divided into classes.

**Table 6:** Estimated relative preference weights for the 2 classes.

Attribute and levels			Class 1 (n=77)					Class 2 (n=37)		
			Coefficient (SE)		P value		Coefficient (SE)			P value
**Treatment modality**										
	VR^a^ only	0.02171 (0.07483)		.77		1.84767 (0.17233)			<.001^b^	
	Medication only	–0.00327 (0.07666)		.97		–1.00383 (0.14776)			<.001^b^	
	Counseling only	–0.01844 (0.07641)		.81		–0.84383 (0.14531)			<.001^b^	
**Therapy duration**										
	6 months	0.13392 (0.09565)		.16		–0.14452 (0.17659)			.42	
	12 months	0.20073 (0.09506)		.04^c^		–0.13237 (0.17046)			.44	
	18 months	–0.06778 (0.09688)		.49		0.29698 (0.17683)			.09	
	1 Lifetime	–0.26687 (0.09761)		.008^c^		–0.02009 (0.17657)			.91	
**Perceived remission rate**										
	35%	–0.16277 (0.11263)		.15		0.37689 (0.19847)			.06	
	40%	0.06315 (0.11437)		.58		0.25085 (0.20222)			.22	
	45%	–0.03595 (0.11223)		.75		–0.13115 (0.20607)			.53	
	50%	0.05105 (0.11274)		.65		0.01580 (0.20174)			.94	
	55%	0.08453 (0.11305)		.46		–0.51237 (0.21644)			.02^c^	
**Probability of adverse events**										
	0%	0.79611 (0.07858)		<.001^b^		–0.05400 (0.13866)			.70	
	30%	–0.07486 (0.07208)		.30		–0.01935 (0.14090)			.89	
	50%	–0.72125 (0.07926)		<.001^b^		0.07336 (0.14025)			.60	
**The monthly cost of treatment^d^**										
	¥500	0.29184 (0.11329)		.01^c^		0.99343 (0.20344)			<.001^b^	
	¥1000	–0.01184 (0.11108)		.91		0.64172 (0.20413)			.002^c^	
	¥1500	–0.01581 (0.11449)		.89		0.02437 (0.20354)			.90	
	¥2000	–0.18010 (0.11332)		.12		–0.39729 (0.21176)			.06	
	¥2500	–0.08408 (0.11113)		.45		–1.26223 (0.23087)			<.001^b^	

^a^VR: virtual reality.

^b^P<.001 indicates highly statistical significance.

^c^P<.05 indicates statistical significance.

^d^A currency exchange rate of ¥1 (Chinese yuan)=US $0.15 is applicable.

**Table 7:** Odds ratios and CIs of attribute levels in the 2 classes.^a^

Attribute and levels			Class 1 (n=77), odds ratio (95% CI)		Class 2 (n=37), odds ratio (95% CI)
**Treatment modality**					
	VR^b^ only	Reference		Reference	
	Medication only	0.975 (0.842-1.129)		0.058 (0.041-0.081)	
	Counseling only	0.961 (0.827-1.116)		0.068 (0.051-0.090)	
**Therapy duration**					
	6 months	Reference		Reference	
	12 months	1.069 (0.887-1.288)		1.012 (0.725-1.414)	
	18 months	0.817 (0.676-0.988)		1.555 (1.100-2.199)	
	Lifetime	0.670 (0.553-0.811)		1.133 (0.801-1.601)	
**Perceived remission rate**					
	35%	Reference		Reference	
	40%	1.253 (1.002-1.568)		0.882 (0.593-1.310)	
	45%	1.135 (0.911-1.415)		0.602 (0.402-0.901)	
	50%	1.238 (0.993-1.545)		0.697 (0.469-1.035)	
	55%	1.281 (1.026-1.598)		0.411 (0.269-0.628)	
**Probability of adverse events**					
	0%	Reference		Reference	
	30%	0.419 (0.363-0.482)		1.035 (0.785-1.365)	
	50%	0.219 (0.188-0.256)		1.136 (0.863-1.495)	
**The monthly cost of treatment^c^**					
	¥500	Reference		Reference	
	¥1000	0.738 (0.594-0.918)		0.703 (0.472-1.050)	
	¥1500	0.735 (0.587-0.920)		0.379 (0.255-0.565)	
	¥2000	0.624 (0.500-0.779)		0.249 (0.164-0.377)	
	¥2500	0.687 (0.552-0.854)		0.105 (0.067-0.165)	

^a^The table presents general results of the multinomial logit model. Data on preferences of respondents from the 2 classes for psychotherapy intervention attributes are reported (N=114).

^b^VR: virtual reality.

^c^A currency exchange rate of ¥1 (Chinese yuan)=US $0.15 is applicable.

**Figure 7:**
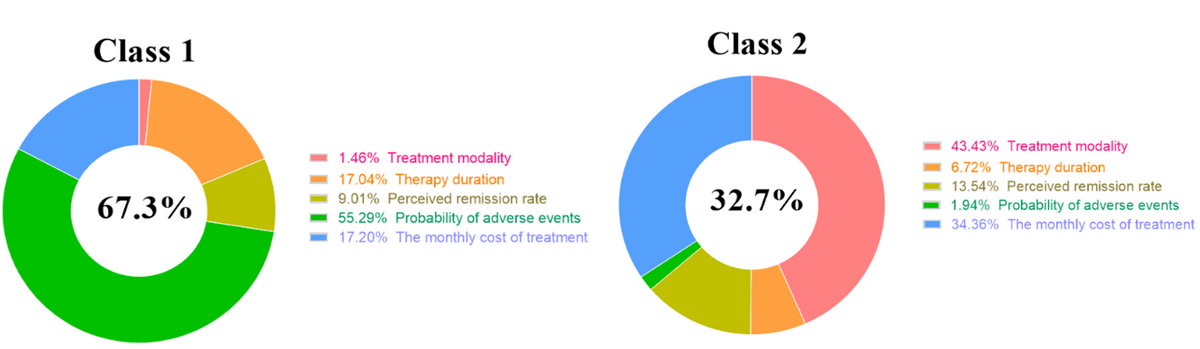
Percentage importance of attributes in the latent class condition.

**Figure 8:**
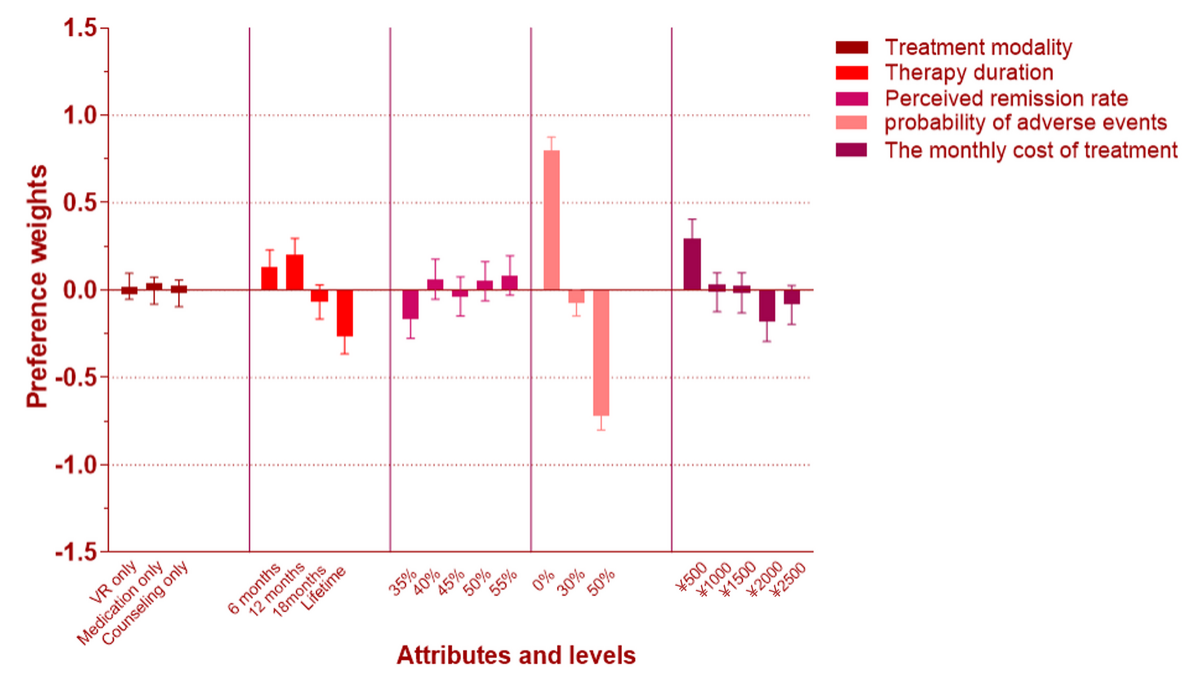
Latent class percentage weights in class 1. VR: virtual reality.

For class 1, the attribute “Probability of adverse events” was the most important factor for respondents with a percentage importance value of 55.29%, followed by “The monthly cost of treatment” and “Therapy duration,” with percentage importance values of 17.20% and 17.04%, respectively (Figure 7). Meanwhile, the span of percentage weights of “Probability of adverse events” was obvious (Figure 8), ranging from −0.72 to 0.79. The span of the attribute “The monthly cost of treatment” ranged from 0.29 to −2.33. The preference weight of “The monthly cost of treatment” decreased with increasing expenses. In addition, the OR of “50%” of the attribute “Probability of adverse events” was 0.219 (95% CI 0.188-0.256), indicating that the majority of patients preferred to pay more attention to the safety of treatment modality with respect to all the levels of the attribute “The monthly cost of treatment” when compared with the reference level “¥500” of <1. At the same time, the OR (Table 7) of the levels of the attribute “Therapy duration” decreased with treatment time, indicating that peoples’ preference for this attribute increased as treatment time decreased.

**Figure 9:**
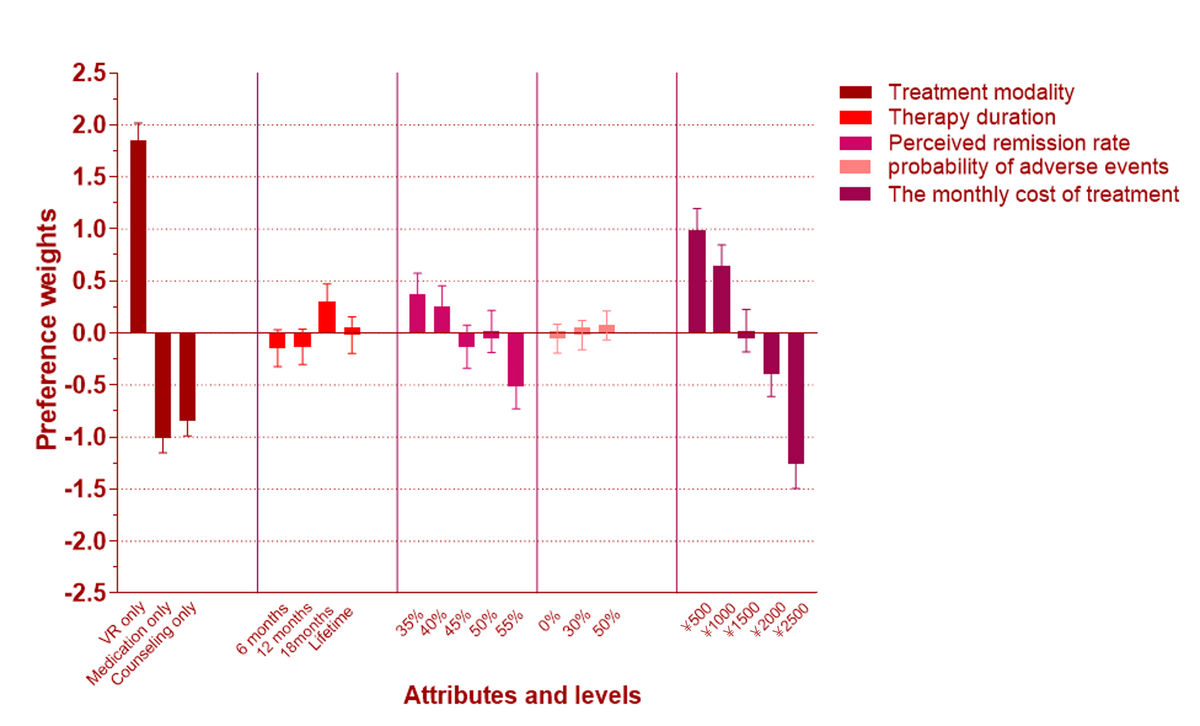
Latent class percentage weights in class 2.

For class 2, the attributes “Treatment modality” and “The monthly cost of treatment” were relatively important; for respondents in this class, the percentage importance values were 43.43% and 34.36%, respectively (Figure 7). For the attribute “Perceived remission rate,” the percentage importance value was 13.54% (Figure 7). The span of the percentage weights of these 2 attributes is presented in Figure 3. The percentage weights for the attributes “Treatment modality” and “The monthly cost of treatment” ranged from −1.003 to 1.847 and −1.26 to 0.073, respectively (Figure 9). In addition, the OR of “Medication only” of the attribute “Treatment modality” was 0.058 (95% CI 0.041-0.081), which was <1, indicating that the VR method appeared to be the best of the 3 methods. The OR of the levels of “Therapy duration” were all >1, and it was different from class 1. Meanwhile, the OR continued to decrease with the increase in the monthly cost of treatment, which was the same condition as that found in the previous class.

For these 2 latent classes, the attribute “Probability of adverse events” was the most preferred factor in class 1, whereas “Treatment modality” was the most preferred factor in class 2. There were 74 and 34 participants in class 1 and class 2, respectively. Class 1 has a similar number of male (39/74, 52.7%) and female participants (35/74, 47.3%), whereas the number of male participants (21/34, 61.8%) is nearly 2 times than that of female participants (13/34, 38.2%) in class 2. In addition, nearly two-thirds of participants in class 1 were medical students (48/74, 64.9%), with nonmedical students comprising only a minority (26/74, 35.1%). By contrast, the numbers of medical and nonmedical students in class 2 were almost even (18/34, 52.9%, and 16/34, 47.1%, respectively).

## Discussion

### Principal Findings

Through the investigation of 5 different attributes and several levels in this survey, our preliminary results indicate that the treatment modality and the probability of adverse events will interfere with people’s choice of therapy. Although a host of people are willing to choose VR as the treatment method instead of others, the treatment modality is not the element that most concerns them. However, the influential weight of the treatment modality is second only to the probability of adverse events, especially for college students who have used VR devices. Although the VR headset did not cause severe adverse reactions immediately, such as vomiting and diarrhea, the disadvantages of overweight devices and dizziness during use should not be ignored.

People who have previously used a VR headset and have had uncomfortable reactions after experiencing VR may have different preferences than those reported in this survey. After we finish the DCE experiment for university students, we hope to carry out a clinical VR experiment for the treatment of depression. Although VR has many advantages in the treatment of mental diseases, some problems may occur when VR is actually applied in the treatment of severe psychological depression, such as a lack of willingness to wear VR glasses. We are willing to compare the treatment of VR in clinical patients to better improve the use of VR in stress counseling and depression treatment.

Latent class analysis results showed that most participants in class 2 would consider “Treatment modality” as the most important attribute. As the number of male participants is almost 2 times more than that of female participants in class 2, this result may be due to a more receptive attitude toward VR technology and VR therapy among male participants, compared with female participants. Male students are also more likely to play computer games and VR games in daily life, whereas women might pay more attention to the details of the equipment, such as equipment weight, dizziness, and air permeability. In addition, our results revealed that the majority of participants in class 1 would consider the “Probability of adverse events” as the most important attribute. This result is possibly due to the prominent number of medical students in class 1. Because of the specialty of their major, it is common that participants from clinical medicine and related majors would understand more about the side effects of antidepressants and be more aware of the complications of using VR technology. Thus, it is not surprising that most participants in class 2 chose “Probability of adverse events” as the most important attribute.

The results of the DCE experiment in this study are helpful for doctors and researchers to conduct further analysis, so they can devise better clinical strategies for the treatment of depression. We separated different treatment methods and compared the effects of individual choices, such as just focusing on medical therapy for depression and analyzing the influence from different perspectives (time and cost). At the same time, our experimental results could promote VR technology for the treatment of depression and provide reference and guidance for individuals and hospitals that are willing to use VR treatment.

Our study’s results showed that individuals prefer VR technology as a psychological intervention method for treating psychological disorders (such as depression) and easing their anxiety. There are several studies that might support our findings [30,31,64,65]. First, an article suggested that a virtual and peaceful environment with relaxing music could make the participants feel like they are in a realistic soothing environment [64]. Second, the research done by Ioannou et al [65] stated that VR could effectively reduce these symptoms in different contexts and with regard to different diseases, including cancer. Another paper suggested that VR has good clinical potential to reduce the severity of depression [30]. A study conducted by Schleider et al [31] revealed that immersive technology, such as VR headsets, may be an efficient strategy for reducing adolescent depressive symptoms. According to the aforementioned studies, the comfortable feeling that VR technology can provide and its effectiveness in treating depression may explain the participants’ preference for using VR technology in treating psychological disorders.

Indeed, most other VR depression experiments are focused on the methodology and VR content making more than the university students’ depression therapy. By contrast, participants’ preferences in our results, such as “Probability of adverse events” and “Treatment modality” from the DCE, could help doctors adopt some new strategies with VR headsets for depression therapy. Besides, in the university environment, the mental health treatment center and psychological counseling room in the school could use VR applications to prevent depression and anxiety for students. Another similar study used VR to enhance employees’ mental health and work performance [66].

### Limitation

There are some limitations to this study. First, the new high technocracies that involve VR may influence university students’ preferences. In addition, participants with knowledge of drugs (antidepressants), such as selective serotonin reuptake inhibitors and serotonin and norepinephrine reuptake inhibitors, may cause bias in the preferred choice in the questionnaire [67]. All of these factors will cause the experimental process to become more complex. We will continue to perform a variety of mixed treatment experiments in the future to further accurately analyze the effects of different combinations of treatment methods for depression. At the same time, we will also focus on more parameters, such as gender, major, and age, in depression therapy. For example, medical students will have some bias in depression treatment compared with other students.

### Conclusion

People placed significant preference on VR technology’s psychological intervention methods, which indicates that VR may have a potential market for treating psychological problems, especially for university students. However, most participants were concerned about the side effects and treatment modality of the VR technology, and these 2 attributes need to be considered when designing treatment plans for individuals. This study can guide policies relevant to the development of the application of VR technology in the field of psychological pressure and depression treatment. At the same time, doctors can consider adopting multiple methods, such as VR plus counseling, in clinical applications.

## Abbreviations

AIC3: Akaike information criterion 3
BIC: Bayesian information criteria
DCE: discrete choice experiment
OR: odds ratio
UESTC: University of Electronic Science and Technology of China
VR: virtual reality
